# Petersen Hernia After Abdominoplasty: A Provocative Factor or a Coincidence?

**DOI:** 10.7759/cureus.45014

**Published:** 2023-09-11

**Authors:** Leonor Caixeiro, João Varanda, João Morais, António Ferreira, Leonor Rios

**Affiliations:** 1 Plastic and Reconstructive Surgery, Centro Hospitalar V.N.Gaia/Espinho, Vila Nova de Gaia, PRT; 2 General Surgery, Centro Hospitalar V.N.Gaia/Espinho, Vila Nova de Gaia, PRT; 3 Plastic and Reconstructive Surgery, Hospital da Prelada, Porto, PRT

**Keywords:** roux-en-y gastric bypass, bariatric surgery, acute abdomen, petersen hernia, abdominoplasty

## Abstract

Abdominal contouring procedures are frequently performed following massive weight loss after a Roux-en-Y gastric bypass. This last procedure can be associated with late complications like the development of internal hernias (e.g., Petersen hernia). The purpose of this article is to present a case of Petersen hernia after complete abdominoplasty.

A 42-year-old female presented to the emergency department with acute abdominal pain three days after undergoing a complete abdominoplasty following massive weight loss post laparoscopic gastric bypass (Roux-en-Y). The patient was diagnosed with a mechanical small bowel obstruction, likely due to an internal hernia (Petersen hernia) and underwent surgical correction.

Gastric bypass surgery may be associated with small bowel obstruction from internal herniation. A sudden rise in abdominal pressure, as occurs with abdominoplasty with rectus plication and subsequent use of compression garments, may result in strangulation and intestinal ischemia. A high degree of suspicion of intra-abdominal complications after abdominoplasty is essential, particularly in the setting of post-bariatric surgery.

## Introduction

Abdominoplasty is one of the most common aesthetic procedures performed worldwide [1]. Abdominal contouring procedures are frequently done after bariatric surgery and massive weight loss. The patients who underwent Roux-en-Y gastric bypass are more prone to develop small bowel obstruction from many causes [2]. We present a case report of a patient with a previous laparoscopic Roux-en-y gastric bypass who presented with acute abdominal pain following abdominoplasty. Surgical exploration showed a Petersen hernia - a type of internal hernia that occurs in the potential space posterior to a gastrojejunostomy. This hernia is caused by the herniation of intestinal loops through the defect between the small bowel limbs, the transverse mesocolon, and the retroperitoneum, after any type of gastrojejunostomy [3-5]. To the best of our knowledge, this is the first report of intestinal ischemia due to Petersen hernia in the early post-operative course of abdominoplasty in the literature.

The purpose of this article is to present a rare case of intra-abdominal complications after abdominoplasty related to previous laparoscopic gastric bypass (Roux-en-Y) - Petersen internal hernia. The symptoms and signs, management, and the possible etiology of this disorder are discussed.

## Case presentation

**Figure 1:**
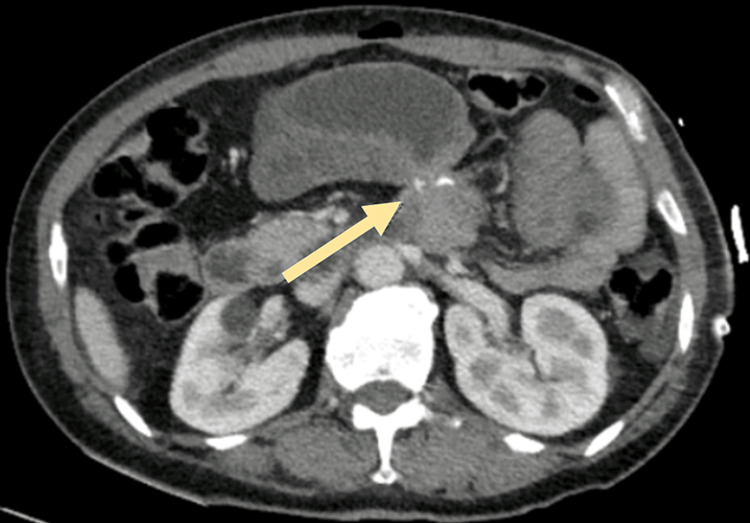
CT scan showing the abrupt stop sign in a proximal jejunal loop.

A 42-year-old female presented to the emergency department with intermittent diffuse abdominal pain with radiation to the left flank, nausea, and vomiting within one day of evolution. No genitourinary symptoms were reported. No bowel movements were reported in the previous four days. Three days before, the patient underwent an abdominoplasty with rectus plication and the procedure was uneventful. She has a history of laparoscopic gastric bypass (Roux-en-Y), 2,5 years before, after which the patient complained of chronic abdominal discomfort related to food intake. Blood tests showed normal hemoglobin, without leukocytosis, with mild elevation of C-reactive protein (2,7). CT Scan showed gastric, duodenum, and jejunal distension; with parietal thickening, wall heterogeneity, and mucosal hypercaptation in the proximal jejunal loop; a stop point in the proximal jejunum and rotation of mesenteric vessels, suggesting an internal hernia (Figure 1).

A diagnosis of mechanical small bowel obstruction, likely due to an internal hernia (Petersen hernia) was made. The patient underwent a median laparotomy. Lysis of adhesions and mobilization of the great epiploon was performed. Retrieval of the proximal intestinal loops from the Petersen space was accomplished. About 30 cm of necrotic proximal jejunum in the biliopancreatic limb was removed and a lateral-lateral jejunojejunal anastomosis was carried out. Subsequently, the Petersen space was closed with Vicryl 3/0 and the abdominal wall closed as usual. She underwent a course of antibiotherapy (ciprofloxacin and metronidazol) and was discharged five days later with a resolution of the symptoms. A subsequent CT scan showed no recurrence of the internal hernia.

## Discussion

**Figure 2:**
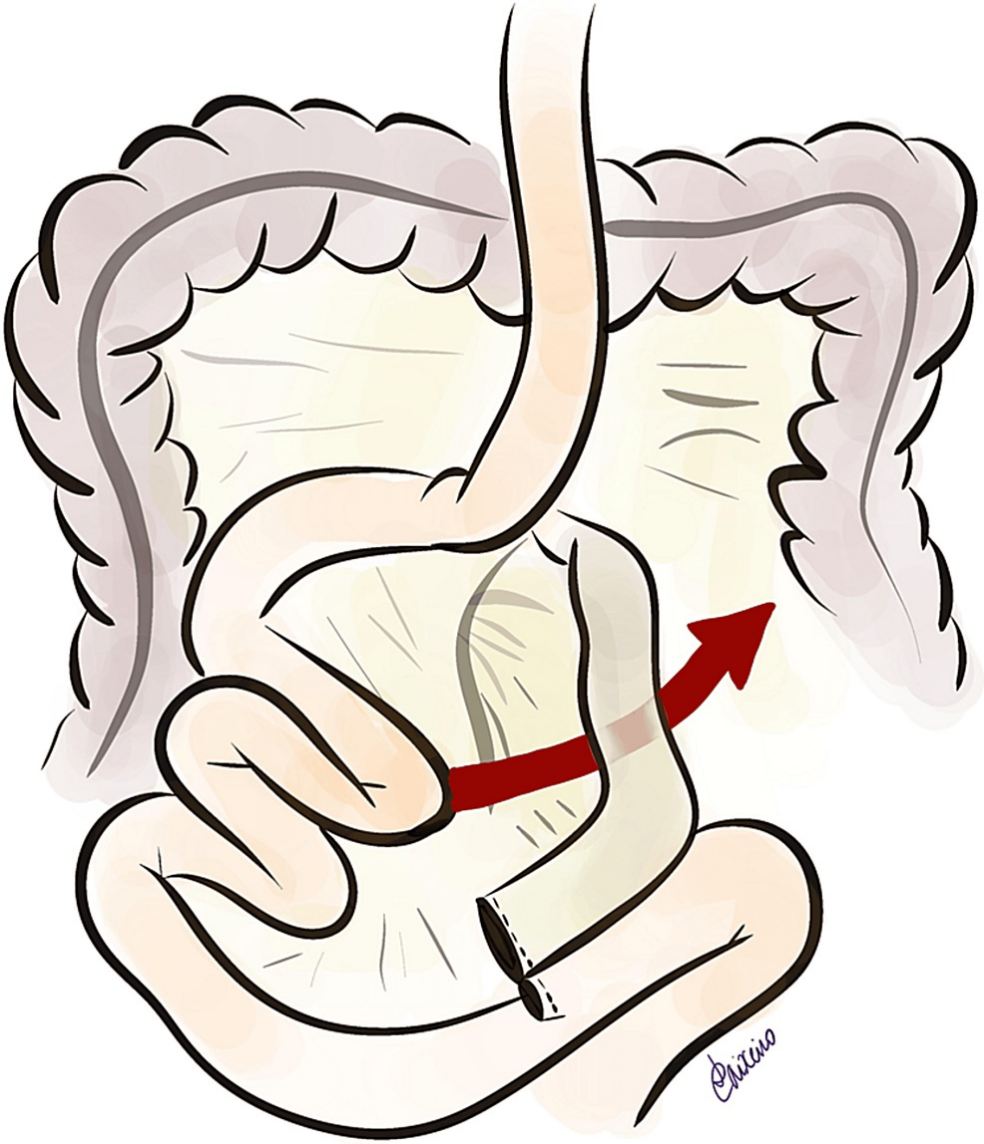
Potential space for internal herniation following Roux-en-Y gastric bypass surgery - Petersen hernia, through the posterior aspect of the mesentery of the Roux limb. Image Credits: Leonor Caixeiro

The Petersen internal hernia (Figure 2) used to be considered rare but is becoming more frequent with the exponential growth in laparoscopic gastric bypass for the treatment of obesity [6-13]. In laparotomy, the incidence has been reported to be 1%-5% [6] and slightly higher in laparoscopic surgery (3.1%-9.7%) [7,13,14] probably because the least invasive procedure results in less adhesions and more mobile intestinal loops [15-17].

Patients with a history of gastric bypass surgery have a high incidence of small bowel obstruction from internal herniation, which is associated with non-specific upper abdominal pain, nausea, and anorexia [18]. The fact that our patient had recurrent abdominal discomfort related to food intake may suggest that there was herniation of the jejunum loops through the Petersen space previous to the abdominoplasty. It is well documented the rise of abdominal pressure after abdominoplasty (due to tight abdominal closure, plication, binders, etc.) [19]. When there is a sudden rise in abdominal pressure, these internal hernias may aggravate, resulting in strangulation and intestinal ischemia [20]. We suspect that the rise in abdominal pressure may have exacerbated the herniation of the intestinal loops.

Abdominal pain in the setting of an early post-operative course of abdominoplasty may have a broad differential diagnosis. The most evident cause may be the pain associated with abdominoplasty by itself. When it is associated with obstipation, a postoperative ileus may also be a frequent cause. If abdominal guarding, rigidity or rebound tenderness, intra-abdominal complications must be sought like a stitch through the bowel, internal hernias, adhesions, and volulus. Even common inflammatory causes of acute abdomen may be coincidently present like appendicitis or cholecystitis. An adequate evaluation by an experienced surgeon is essential to diagnose and treat these difficult cases.

## Conclusions

Although a rare case is described in this report, a high degree of suspicion of possible intra-abdominal complications after abdominoplasty is essential, particularly in the setting of post-bariatric surgery. The plastic surgeon should be aware of this gastric bypass complication when performing body contouring procedures.
